# Actin polymerization is not required for the fast block to polyspermy in the African clawed frog, *Xenopus laevis*

**DOI:** 10.17912/micropub.biology.000365

**Published:** 2021-02-09

**Authors:** Maiwase Tembo, Monica L Sauer, Bennett W Wisner, David O Beleny, Marc A Napolitano, Anne E Carlson

**Affiliations:** 1 Department of Biological Sciences, University of Pittsburgh

## Abstract

Fertilization of an egg by multiple sperm presents one of the earliest and most prevalent obstacles to successful reproduction. Eggs employ multiple mechanisms to prevent sperm entry into the nascent zygote. The fast block to polyspermy uses a depolarization to inhibit sperm entry. For some external fertilizers, fertilization and the fast block require actin polymerization. Here we explored whether the fast block to polyspermy in the external fertilizer, *Xenopus laevis*, requires actin polymerization. Inseminating in the presence of inhibitor cytochalasin B, here we demonstrate that actin polymerization is not required for the fast block to polyspermy in *X. laevis*.

**Figure 1:**
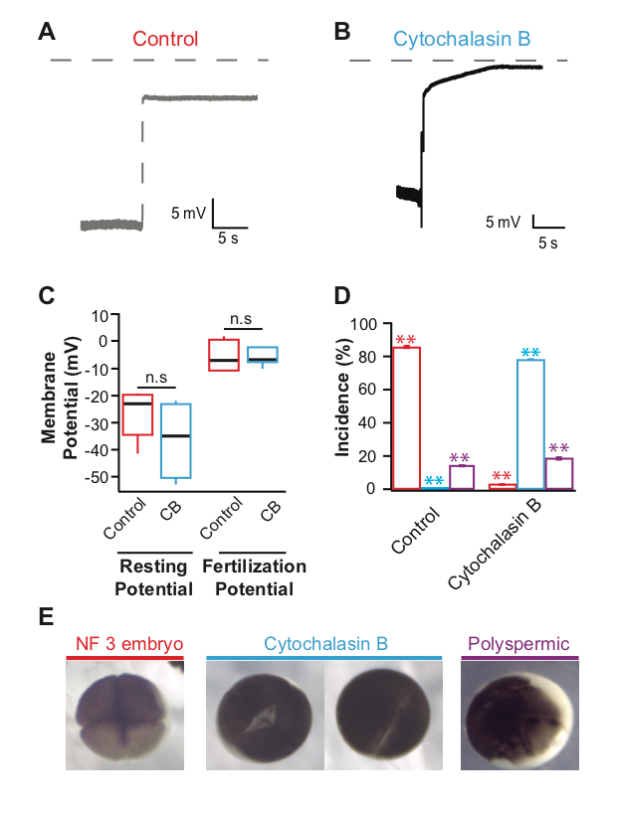
Representative whole-cell recordings made during fertilization in control conditions **(A)** or 10 µg/ml cytochalasin B **(B)**. Grey dashed lines denote 0 mV. Tukey box plot distributions of the resting (n = 10) and fertilization (n = 4) potentials **(C)**. **(D)** Average percentage (± s.e.m.) of embryos inseminated under control conditions (red) or with 10 µg/ml cytochalasin B (blue), that developed normally to the 4-cell (NF 3) stage, with incomplete cytokinesis, or with abnormal cleavage furrows (n = 14), colors correspond to images shown in E. **(E)** Representative images of a NF 3 embryo, two cytochalasin B treated embryos with incomplete cytokinesis and a polyspermic embryo with abnormal cleavage furrows. *** denotes *P* < 0.001 and n.s denotes a *P* > 0.05 as determined by Student’s t-tests with the Bonferroni correction.

## Description

For most animals, polyspermic fertilization of eggs is embryonic lethal (Bianchi and Wright, 2016) and must be prevented. Eggs of externally fertilizing animals employ a mechanism known as the fast block to polyspermy to inhibit fertilization by more than one sperm (Bianchi and Wright, 2016). Within seconds of fertilization, the fast block activates a depolarization of the egg membrane (Wozniak *et al.*, 2018). Sperm can bind to, but not enter, depolarized eggs (Jaffe, 1976). In addition to becoming depolarized, the egg also extends polymers of actin to bring in sperm at fertilization in externally fertilizing animals such as sea urchins, starfish, and zebrafish (Silvestre and Tosti *et al.* 2010). Because actin plays a prominent role bringing sperm into the of eggs of many externally fertilizing animals, we hypothesized that actin polymerization is needed for the fast block to polyspermy in the African clawed frog, *Xenopus laevis*.

Actin polymerization is inhibited by the application of the potent drug cytochalasin B (MacLean-Fletcher and Pollard 1980). In sea urchin eggs, the actin filaments disappeared after incubation in cytochalasin B for at least 5 minutes (Chun, *et al.*, 2014). We sought to evaluate if at similar concentrations, the inhibition of actin polymerization would prevent the fast block to polyspermy in *X. laevis* eggs. To address our question, we conducted whole cell recordings during fertilization of *X. laevis* eggs in the presence or absence of the potent actin polymerization inhibitor, cytochalasin B. Under control conditions or in the absence of cytochalasin B, we observed normal depolarizations with fertilization (Figure 1A) (Wozniak *et al.*, 2018). *X. laevis* eggs inseminated in 10 µg/ml cytochalasin B also depolarized (Figure 1B). The shapes of the depolarizations are slightly different, as observed in recordings made in control conditions. This concentration of cytochalasin B was chosen because it completely inhibited sperm entry into eggs of another external fertilizer, zebrafish (Wolenski and Hart, 1988). Importantly, sperm entry is facilitated by the depolarization of the membrane in the external fertilizer, the sea urchin (McCulloh, *et al.* 1987). The depolarization of the sea urchin egg membrane is required for both sperm entry and the fast block to polyspermy (McCulloh, *et al.* 1987). Our observation that *X. laevis* eggs depolarized despite the inhibition of actin polymerization suggested that although *X. laevis* is an external fertilizer like sea urchins, the processes involved in sperm entry and depolarization are different.

To examine if Cytochalasin B changed the egg’s electrical properties in a way that would affect the depolarizations recorded, we assessed the time before depolarization and egg membrane potentials. Here, *X. laevis* eggs were incubated in cytochalasin B for an average of 18.6 min before depolarizations were recorded. Cytochalasin B application did not change the resting or fertilization potentials; eggs inseminated under control conditions had an average resting potential of -26.6 ± 2.9 mV (n = 10) and depolarized to 5.1 ± 1.0 mV (n = 10) which was similar to eggs inseminated in cytochalasin B whose, resting and fertilization potentials were -36.1 ± 7.2 mV (n = 4) and -5.4 ± 3.1 mV (n = 4) (Figure 1C). These data indicate that cytochalasin B application did not alter the fertilization-evoked depolarization. This is unlike the polyspermy block findings observed for the monospermic external fertilizer, the marine bivalve mollusk, *Spisula*, whose incidence of polyspermy increased with high concentrations of cytochalasin B (Ziomek and Epel 1975).

To determine whether 10 µg/ml cytochalasin B was acting on the *X. laevis* egg actin, we assessed cleavage furrow-development of the embryos. To form cleavage furrows, *X. laevis* eggs must undergo F-actin reorganization (Noguchi and Mabuchi, 2001), a process requiring actin polymerization. Furrow formation resulting in symmetric cleavage furrows with deep cleavages is associated with successful fast blocks to polyspermy and monospermic fertilization (Noguchi and Mabuchi, 2001). In 14 independent trials, sperm were added to groups of 10-50 eggs in 0 or 10 µg/ml cytochalasin B. 90 minutes after sperm addition, appearance of cleavage furrows was assessed for embryos in each condition. Whereas 86.6 ± 0.7% (n = 14) of eggs inseminated under control conditions developed symmetrical cleavage furrows and reached the 4-cell (NF 3) stage, 78.1 ± 3.4% (n = 14) eggs inseminated in cytochalasin B displayed incomplete cytokinesis (Figure 1E). When actin polymerization was completely inhibited, the zygotes displayed a white band of newly added plasma membrane without contraction of the egg cortex. It is possible that this concentration of cytochalasin B may effectively inhibit the actin polymerization required for cytokinesis and yet be ineffective at inhibiting the polymerization required for fertilization-evoked depolarization. However, cytochalasin B disruption of cytokinesis supports that the compound effectively inhibited actin polymerization in the *X. laevis* eggs. Not only does the egg remain fertilizable in the presence of cytochalasin B, it is capable of initiating the fast block to polyspermy unlike sea urchins or the bivalve mollusk *Spisula*. Because the drug acts on the microfilament system of the egg, these data could have wider implications for how we think about *X. laevis* fertilization. Together, the data presented here suggest that actin polymerization is not required for the fast block to polyspermy.

## Methods

All animal protocols were conducted using accepted standards of humane animal care, approved by the Animal Care and Use Committee at the University of Pittsburgh. Adult *X. laevis* frogs were commercially purchased (Nasco) and housed at 18°C in 12/12 hr light and dark cycle. Ovulation was induced by injection of 1,000 IU of hCG into the dorsal lymph sac. Frogs were housed at 14°C for 14-16 hours after injection. Females typically began laying eggs 0-2 hours after their transfer to room temperature (22^o^C). Eggs were collected onto dry petri dishes and were used within 10 min.

Testes were collected from mature *X. laevis* males following euthanasia by a 30-min immersion in 3.6 g/L tricaine-S, pH 7.4. To create a sperm suspension, 1/10 of a testis was minced in MR/3. Eggs were inseminated by pipetting the sperm suspension onto of the eggs bathed in MR/3. Dissected testes were stored at 4^o^C in L-15 medium and used within 5 days.

Modified Ringer’s (MR) solution used was composed of (in mM): 100 NaCl, 1.8 KCl, 2.0 CaCl_2_, 1.0 MgCl_2_, and 5.0 HEPES, pH 7.8. The MR was filtered using a sterile, 0.2-µm polystyrene filter. For fertilization-evoked depolarizations, eggs were inseminated in MR diluted to 20% MR (MR/5) for control conditions or in MR/5 with cytochalasin B added. After fertilization-evoked depolarizations were made for fertilization experiments, embryos were set aside to develop in MR diluted to 33% MR (MR/3). For developmental assays, embryos were incubated in MR/3 for control conditions or in MR/3 with cytochalasin B added.

Fertilization-evoked depolarizations were recorded as previously described (Wozniak *et al.* 2018). Briefly, recordings were made in the whole-cell using TEV-200A amplifiers (Dagan Co.) and digitized by Axon Digidata 1550A (Molecular Devices). The data were collected with pClamp Software (Molecular Devices) at a rate of 5 kHz. Pipettes made from borosilicate glass were 8–20 MΩ resistance and filled with 1 M KCl. Membrane potentials were quantified ∼10 s before (resting) and after (fertilization) the depolarization. Data were analyzed in Excel (Microsoft) and IGOR (Wavemetrics).

Approximately 10-50 eggs were inseminated in each experimental condition and then assessed for development based on the appearance of cleavage furrows (Figure 1E). Developmental phenotypes for each frog’s eggs were scored out of the total number of eggs fertilized and developed in each condition on each experimental day. All experiments were conducted at 22^o^C.

## Reagents

**Table:** 

**Reagent**	**Source**	**Identifier**
Cytochalasin B	Thermo Fisher	PubChem CID 5311281
Chorulon hCG	Henry Schein	Cat. 28938
L-15 medium	Sigma	Cat. L1518-500ml
Tricaine-S	Thermo Fisher	Cat. NC0342409
